# Coding-complete genome sequence of a bovine viral diarrhea virus isolate NX2023 from a calf in China

**DOI:** 10.1128/mra.00169-24

**Published:** 2024-06-25

**Authors:** Wei Liu, Junjun Shao, Haiyan Huang, Yijia Liu, Huichen Guo, Huiyun Chang, Jiandong Wang, Gao Shandian

**Affiliations:** 1 Gansu Province Research Center for Basic Disciplines of Pathogen Biology, State Key Laboratory for Animal Disease Control and Prevention, Lanzhou Veterinary Research Institute, Chinese Academy of Agricultural Sciences, Lanzhou, Gansu, China; 2 Institute of Animal Science, Ningxia Academy of Agricultural and Forestry Sciences, Yinchuan, China; Katholieke Universiteit Leuven, Leuven, Belgium

**Keywords:** bovine viral diarrhea virus, coding-complete genome sequence, subgenotype

## Abstract

The coding-complete genome sequence of bovine viral diarrhea virus (BVDV) isolate NX2023 that originated from a calf in China was determined. Phylogenetic analysis showed that the NX2023 strain belongs to the BVDV-1d subgenotype.

## ANNOUNCEMENT

Bovine viral diarrhea virus (BVDV) mainly infects cattle and can lead to abortion, diarrhea, hemorrhages, and even death (1). It belongs to the *Flaviviridae* family, *Pestivirus* genus. Within the genus, proposed species include *Pestivirus* A through *Pestivirus* K (2). Members of *Pestivirus* A (BVDV-1), *Pestivirus* B (BVDV-2), and *Pestivirus* H (BVDV-3) mainly cause diseases in cattle (3, 4). In this study, we reported the coding-complete genome sequence of BVDV isolate NX2023 that originated from a calf in China.

A blood sample was collected from a calf with respiratory symptoms of coughing and severe nasal discharge in Ningxia Hui Autonomous Region, China in 2023. BVDV antigen was detected in the sample by HerdCheck BVDV antigen/Serum Plus Test Kit (IDEXX Laboratories Inc., USA). The blood sample was used for virus isolation in MDBK cells, as described previously (5). Briefly, a total volume of 1 mL blood sample was exposed to three freeze–thaw cycles and adsorbed onto MDBK cells in a T25 flask, followed by incubation at 37°C in a humidified atmosphere of 5% CO_2_ for 2 h. The inoculum was discarded, and the cells were rinsed twice with PBS and then cultured with 5 mL DMEM supplemented with 2% horse serum, 100 IU/mL penicillin, and 100  µg/mL streptomycin. The infected MDBK cells were checked daily for 4 days and blind passaged four times if no cytopathogenic effect (CPE) was observed. The isolate NX2023 was non-cytopathogenic. Total RNA was extracted from the fourth passage culture with a total RNA extraction kit (Solarbio, Beijing, China). Using the primer pairs of US-10/DS-5782 and US-5430/J2-R (6), the 5'-half (5,793 bp) and 3'-half (6,771 bp) genomic DNA fragments were amplified with PrimeScript One Step RT-PCR Kit (TaKaRa, Dalian, China). The RT-PCR reaction was performed at 50°C for 30 min, followed by 35 cycles at 94°C for 30 s, 55°C for 30 s, 72°C for 7 min, and a final extension at 72°C for 10 min. The PCR products were purified with a DNA Gel Extraction Kit (Beyotime Institute of Biotechnology, Shanghai, China) and submitted to Beijing Tsingke Biotech Co., Ltd for sequencing. The BigDye Terminator v3.1 Cycle Sequencing Kit was used for Sanger sequencing on a 3730XL DNA Analyzer with walking primers (Table 1). The obtained sequences were assembled by Lasergene v7.0.

**TABLE 1:** Primer used for sequencing

Primers	Primer sequence (5′−3′)	Positions	Corresponding strand	References
326	TCAACTCCATGTGCCATGTAC	335–355	Antisense	(7)
BD2	TTGTTRTGGTACARRCCGTC	1,043–1,061	Antisense	(7)
P1	ATAGCCCGACACCACTGACA	1,541–1,560	Sense	This Study
P2	CAGAACGCCGCTTGCGGTTA	1,628–1,647	Antisense	This Study
E2F	TGGTGGCCTTATGAGAC	2,254–2,270	Sense	(8)
P7R	CCCATCATCACTATTTCACC	3,595–3,614	Antisense	(8)
P3	AGAGTTCTTACTGGTCGTGGTGG	3,441–3,463	Sense	This Study
P4	CAGGTGGAAGGTTGACATAGC	4,143–4,163	Sense	This Study
DS5782	TGCYTRAAGTCYCCCCTRTTCAT	5,755–5,777	Antisense	(6)
US5430	GACAGAGTATGGYGTCAAGAC	5,403–5,423	Sense	(6)
P5	GAGCATAAGGGTGGTGGC	6,135–6,152	Sense	This Study
P6	ATTGGAGGTGGGTGGTGTCT	7,278–7,297	Antisense	This Study
P7	CAGTATGACGACGCTTTCCA	7,904–7,923	Antisense	This Study
P8	CCTGGAATTATCACAACCTCG	7,808–7,828	Sense	This Study
P9	AGGGATGGACTCAGAAGGAAA	8,358–8,378	Sense	This Study
P10	TGGTGTCGTTCCTGTTGTCA	9,251–9,270	Antisense	This Study
P11	GTCAACGAAGAATCTGGGACT	9,109–9,129	Sense	This Study
P12	CTTTCCTGTTTATTCCTGCTTC	10,516–10,537	Antisense	This Study
P13	AATAGTCAGAGCCCAGACGG	10,341–1,030	Sense	This Study
P14	AGCCTTGTTGCCATCTTTG	11,450–11,468	Antisense	This Study
P15	AAATAACAGAAGGGGATAAAATG	11,312–11,334	Sense	This Study

**Fig 1:**
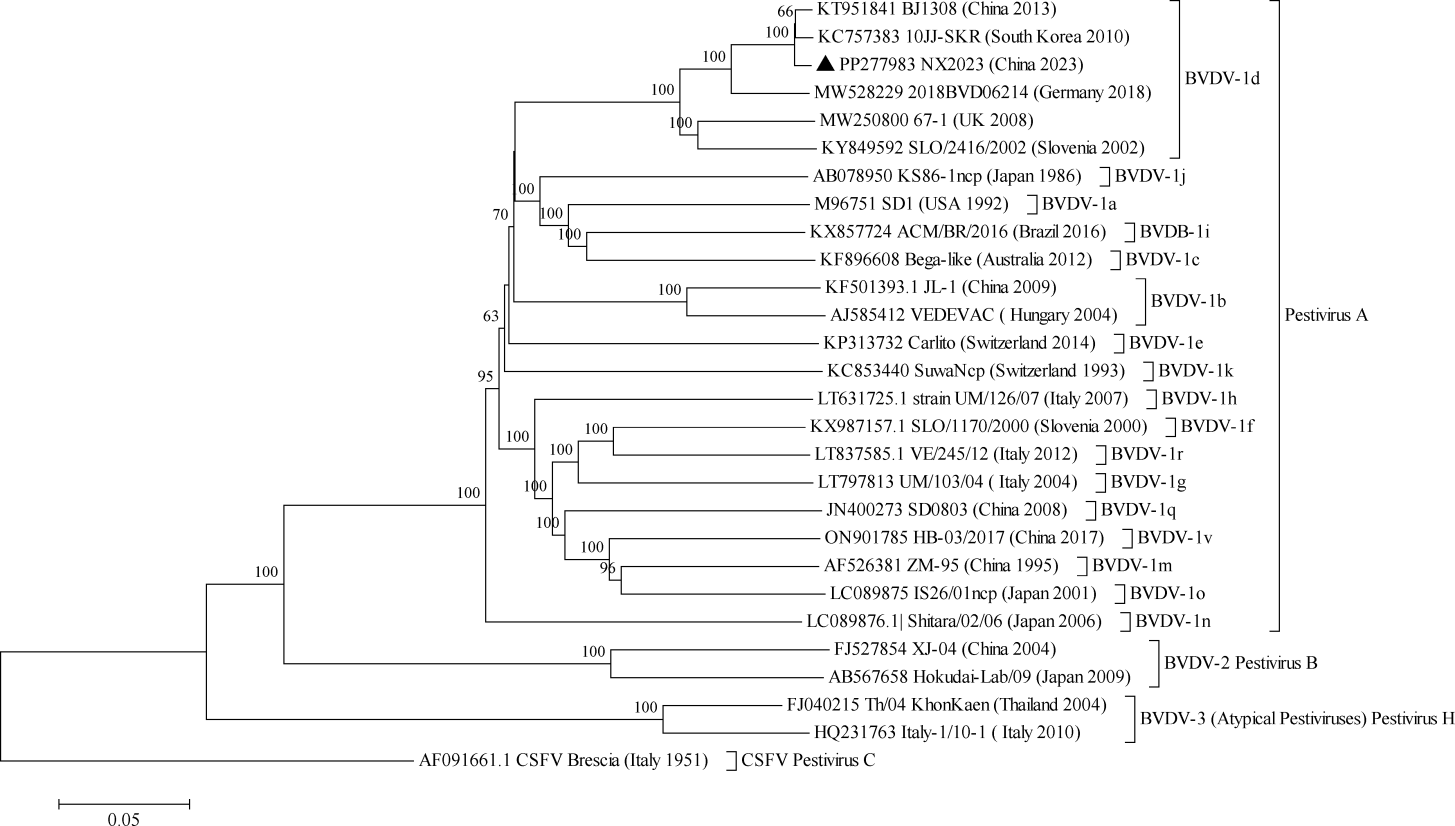
Neighbor-joining phylogeny of BVDV isolates based on coding-complete genome sequences. The phylogenetic tree was constructed using the neighbor-joining method and bootstrap analysis (*n* = 1,000). The reference sequences were selected based on the nomenclature previously proposed (9–15). Bar indicates nucleotide substitutions per site. Sequence alignment was performed using the MUSCLE software with default settings (Gap open penalty: −400; Gap extension penalty: 0). The CSFV was used as an outgroup as described previously (16, 17).

The near-full-length genomic sequence was 12,174 nt in length. It encompasses the polyprotein coding region (11,694 nt), which was flanked by the partially determined 5′UTR (345 nt) and 3′UTR (135 nt). It was 81.53%–98.72% nucleotide identical with genomic sequences of BVDV-1 deposited in GenBank. Phylogenetic analysis demonstrated that NX2023 belonged to the 1d subgenotype (Fig. 1). It was closely related to strains originated from China, the Republic of Korea, and Brazil (MG923683), sharing intra-subgenotype genomic nucleotide identities of 97.40%–98.72%. However, the intra-subgenotype genomic nucleotide identity was as low as 90.53%–94.22% with strains from Germany (MW528229) and the United Kingdom (MW250800), indicating that the BVDV-1d strains are globally diverse.

## Data Availability

The coding-complete genome sequence of BVDV isolate NX2023 has been deposited in GenBank under the accession number PP277983.
